# Scabies: an ancient global disease with a need for new therapies

**DOI:** 10.1186/s12879-015-0983-z

**Published:** 2015-07-01

**Authors:** Jackson Thomas, Greg M Peterson, Shelley F Walton, Christine F Carson, Mark Naunton, Kavya E Baby

**Affiliations:** Faculty of Health, University of Canberra, Bruce, 2601 ACT Australia; Faculty of Health, University of Tasmania, Hobart, 7005 TAS Australia; Faculty of Science; Health, Education and Engineering, University of the Sunshine Coast, Sippy Downs, 4558 QLD Australia; School of Medicine and Pharmacology (M503), The University of Western Australia, Nedlands, 6009 WA Australia; Translational Renal Research Group, Harry Perkins Institute of Medical Research, Nedlands, 6009 WA Australia; Charnwood, Canberra, 2615 ACT Australia

**Keywords:** Scabies, Resistance, MDA, Ivermectin, Permethrin, Benzyl peroxide, Treatments, Indigenous, Aboriginal

## Abstract

**Background:**

Scabies is an ancient disease (documented as far back as 2500 years ago). It affects about 300 million people annually worldwide, and the prevalence is as high as about 60 % in Indigenous and Torres Strait Islander children in Australia. This is more than six times the rate seen in the rest of the developed world. Scabies is frequently complicated by bacterial infection leading to the development of skin sores and other more serious consequences such as septicaemia and chronic heart and kidney diseases. This causes a substantial social and economic burden especially in resource poor communities around the world.

**Discussion:**

Very few treatment options are currently available for the management of scabies infection. In this manuscript we briefly discuss the clinical consequences of scabies and the problems found (studies conducted in Australia) with the currently used topical and oral treatments. Current scabies treatment options are fairly ineffective in preventing treatment relapse, inflammatory skin reactions and associated bacterial skin infections. None have ovicidal, antibacterial, anti-inflammatory and/or anti-pruritic properties. Treatments which are currently available for scabies can be problematic with adverse effects and perhaps of greater concern the risk of treatment failure. The development of new chemical entities is doubtful in the near future. Though there may be potential for immunological control, the development of a vaccine or other immunotherapy modalities may be decades away.

**Summary:**

The emergence of resistance among scabies mites to classical scabicides and ineffectiveness of current treatments (in reducing inflammatory skin reactions and secondary bacterial infections associated with scabies), raise serious concerns regarding current therapy. Treatment adherence difficulties, and safety and efficacy uncertainties in the young and elderly, all signal the need to identify new treatments for scabies.

## Background

Scabies has existed for at least 2500 years [1] and currently affects 300 million people annually worldwide. Its listing as a neglected tropical disease by the World Health Organization (WHO) in 2013 [2] recognised the neglect in public and private sector expenditure on this problem, the lack of attention at local, national, and international levels, and the higher incidence of this infection amongst the poor. In Australia it affects about 6 in 10 Aboriginal and Torres Strait Islander children at any given time, more than six times the rate seen in the rest of the developed world [3, 4]. The sequela of scabies predisposes affected children to sepsis and other non-suppurative invasive infections (*e.g.* lymphadenopathy, acute post-streptococcal glomerulonephritis (APSGN) and rheumatic fever) [5]. Outbreaks of APSGN usually coincide with scabies outbreaks, which can contribute to the development of chronic kidney disease and subsequent renal failure in adulthood [6]. It is usually reported in Australian Aboriginal communities, other Oceanic nations (Papua New Guinea, Fiji, Solomon Islands, Vanuatu) [7], and in some parts of India, Chile and Trinidad [5], and is uncommon outside these communities. APSGN outbreaks do not always coincide with scabies outbreaks elsewhere in the developed world. Scabies infestation has a negative impact on the quality of life for infected individuals (similar to that of psoriasis) resulting in substantial stigmatisation and ostracism [8].

In this manuscript we focus on the challenges found with diagnosis and treatment, emerging resistance among scabies mites, and the need for further research in this field to identify new and alternative therapies for the treatment and prophylaxis of scabies.

## Discussion

### Diagnosis

At present there is no accurate means of diagnosing scabies in various clinical settings. Presumptive diagnosis is often made on the basis of clinical signs, and a history of contact with other scabies cases [9]. In practice (especially in resource poor settings), identifying scabies mite from patient’s skin is challenging, and a negative result by even experienced clinical staff, does not rule out scabies. A positive response to therapy cannot exclude the spontaneous disappearance of a skin condition other than scabies, and a negative response to the first-line treatment option does not exclude scabies, especially with growing resistance among scabies mites [9]. Recent findings in this field, *e.g.* serodiagnosis [10] shows promising potential; however, more research is required to evaluate its efficiency in tropical clinical settings.

### Treatments

There are several recommended treatment options for scabies and these have been discussed in detail in a 2010 Cochrane review [11]. There are few obvious safe and effective scabicides currently (Table 1 and 2), and treatment effectiveness can vary between clinical settings [12]. So far, there is no international consensus on the appropriate schedule for scabies treatment, and recommendations in one nation may not be appropriate in others [8]. The first line treatment options are topical agents and require whole body application for many hours duration [13]. Multiple treatment doses are often recommended over days to weeks. Topical or oral antibiotic therapy may be required if secondary skin infection has developed [14]. It has also been advised that close contacts of people with scabies should be treated simultaneously, as they may be infected without yet manifesting symptoms, and so can act as a reservoir of infection [14, 15]. Treating the contacts may prevent reinfection of the index cases following treatment [13]. The logistics to treat all contacts simultaneously are significant as it requires identification and treatment of all contacts of an index case (*e.g.* family members, other coinhabitants, medical and other supporting staff and others who may come in contact with the index cases) [14, 16].

**Table 1:** Evidence of resistance to classical treatments used in scabies management

Study	Indicative cure rate	Drugs and treatment regimen	Comments
**Topical**			
*Benzyl benzoate*			
[45] Moberg *et al.*, 1984	(43 %; 6/14)	benzyl benzoate (22.5 %)	case report
[46] Yonkosky *et al.*, 1990	(12 %; 23/195)	benzyl benzoate (50 %)	case study
[47] Nnoruka *et al.*, 2001	(48 %; 14/29)	benzyl benzoate (22.5 %)	clinical exploratory study
[38] Glaziou *et al.*, 1993	(48 %; 10/21)	benzyl benzoate (10 %)	RCT
*Permethrin*			
[38] Leibowitz, 1993	(0 %; 0/11)	permethrin 5 % cream	un-controlled case study
[40] Fraser, 1994		permethrin	(*in vitro* study)
[48] Walton *et al.*, 2000		"	(*in vitro* study)
[37] Pasay *et al.*, 2006		"	(*in vitro* study)
[39] Pasay *et al.*, 2008		"	(*in vitro* study)
[33] Mounsey *et al.*, 2008		"	(*in vitro* study)
[34] Mounsey *et al.*, 2009		"	(*in vitro* study)
[49] Saqib *et al.*, 2012			quasi clinical study, re-infestation after successful treatment (7 %; 4/60)
[50] Huffam *et al.*, 1997	(0 %; 0/20)	permethrin 5 % cream	crusted scabies
*Sulphur*			
[51] Coskey RJ, 1979	(0 %; 0/1)	sulphur 5 % in an ointment	case report
**Oral**			
*Ivermectin*			
[52] Glaziou *et al.*, 1993	(70 %; 16/23)	single dose, 100 μg/kg	RCT, poor efficacy partly attributed to the lower dose used in the study
[30] Currie *et al.*, 1994	(0 %; 0/1)	two doses, 200 μg/kg	case report, crusted scabies
[33] Currie B.J 1999		five dose regimen, 200 μg/kg	crusted scabies, monthly administration failed to prevent reinfestation
[36] Currie *et al.*, 2004	(0 %; 0/2)	seven doses, 270 μg/kg	reinfestation following seven doses, unpublished observations
[53] Brooks, *et al.*, 2002	(56 %; 24/43)	single dose, 200 μg/kg	results evaluated at 3 weeks post treatment
[33] Mounsey *et al.*, 2008			*In vitro* study
[41] van den Hoek *et al.*, 2008	(0 %; 0/7)		case report
[34] Mounsey *et al.*, 2009			*In vitro* study
[42] Ly *et al.*, 2009		(30 %;16/53) single dose, 150–200 μg/kg	first RCT to report resistance of ivermectin
[43] Rizvi *et al.*, 2011		(78 %; 38/50) single dose, 200 μg/kg	quasi clinical study
[54] Fujimoto *et al.*, 2014	(0 %; 0/1)	6000 μg/week* 3 + 12000 μg/week *3	case report
[49] Saqib *et al.*, 2012			quasi clinical study, re-infestation after successful treatment (7 %; 4/60)
[50] Huffam *et al.*, 1997	(60 %; 12/20)	one-three doses, 200 μg/kg combined with topical scabicide and keratolytic therapy	crusted scabies

**Table 2:** An overview of classical treatments indicated for the management of scabies in Australia

Study	Drugs	Dosage	Treatment regimen	Contraindication	Disadvantages	Comments
	**Topical**					
[55, 56]	Benzyl benzoate	25 % solution	one or several consecutive 24-h applications	pregnant women and infants	burning or stinging, pruritus, dermatitis, convulsions (rare)	In use since 1930s; possible neurological complications with misuse; withdrawn in the European Union due to neurotoxicity concerns
[23, 56–58]	Permethrin	5 % cream (8–14 h) then wash off	apply overnight	infants aged <2 months	mild burning, itching stinging, pruritus, erythema, tingling, persistent excoriation, dystonia (rare), convulsions (rare)	in use since the 1980s; relatively expensive; growing resistance among scabies mites poor compliance reported in mass community intervention programs
[59, 60]	Sulphur	2–10 % precipitate in petroleum base	apply for 24 h, and then wash and reapply repeat applications for 3 days		noxious, malodorous messy; not given as first-line agents; multiple applications required; can cause skin irritation;	has been used for centuries; indicated in infants, pregnant and lactating women; inexpensive
	**Oral**					
[53, 61–63]	Ivermectin	200 μg/kg orally repeated after 1–2 weeks		children aged <5 years.; children <15 kg; pregnant or lactating women	transient side effects: gastrointestinal disorders; pustular rash, cellulitis; abdominal pain, diarrhoea, headache, vomiting, hypotension, toxic epidermal necrosis, mucosal drug eruption, fever, anorexia, lymph node swelling, eosinophilia, pain of joint and muscles, mazzotti reaction	in use since 1980’s (for the mass treatment of onchocerciasis, and filariasis); not approved for the treatment of typical scabies (except in Japan, Brazil, France); only indicated if symptoms persists 3 weeks after application of benzyl benzoate or permethrin; no ovicidal activity, thus repeat treatment is required; one report of increased deaths among elderly patients during scabies outbreak in an institutional setting (1997); there has been considerable criticism on the validity of this report, no other studies have replicated these findings

Most treatments (Table 2) are potentially hazardous and are associated with moderate to severe side effects (*e.g.* secondary eczematisation, oedema, erosions and/or pyoderma) [17–22]. The most frequent complication of topical scabicides is persisting post-scabies eczema (generalised eczematous dermatitis) resulting from irritant effects of the various formulations [23]. These may escalate xerosis and worsen delayed-type eczema. Further, it may be difficult to treat patients with secondary eczematisation, erosions or ulcers using currently available topical scabicidal agents such as permethrin, lindane, benzoyl peroxide and sulphur, as they can cause serious cutaneous and systemic side-effects in addition to the problem of compliance, resulting in poor treatment uptake [24].

Oral ivermectin is not widely available and has not been approved for the management of scabies in many countries [8]. At present, there is some observational evidence of its effectiveness in controlling scabies outbreaks in institutional settings including nursing homes [5]. However; the safety or efficacy of ivermectin, the sole oral therapy available, has not been well-established in the elderly, patients with impaired liver function (potential for toxicity resulting from long elimination half-life [36 h]), children (aged <5 years) and pregnant women, where the blood–brain barrier is incompletely developed in the foetus, raising the potential for neurotoxicity [12]. Because of the drug’s lipophilicity, ivermectin may be poorly distributed in the asteototic stratum corneum of the elderly compared to younger patients, leading to mediocre therapeutic responses in the former patient cohort and requiring combination therapy with topical scabicides [12]. Ivermectin is not ovicidal, does not adequately penetrate the thick egg shell of the scabies mite, and is also ineffective against the younger stages of the parasite (whose nervous system is poorly developed), resulting in delayed therapeutic response [25].

*Mass drug administration (MDA) programs* have been attempted to use ivermectin to control scabies in endemic communities around the world [26]. However, such programs’ superiority over alternative topical treatment is questionable [27, 28]. Ivermectin is indicated in Australia only for crusted scabies or cases of typical scabies when prior topical treatment has failed or has been contraindicated [29]. It is generally recommended that for maximum absorption ivermectin must be given in empty stomach [30], which is a challenge in community-based programs. Ivermectin administration can be labour-intensive since the weight of all patients and the pregnancy status of all women of childbearing age must be determined. Paradoxically, its use is not recommended in those under 5 years of age when these are the most vulnerable group (particularly among Aboriginal and Torres Strait Islander children) [29, 30]. Institutional outbreaks of scabies in aged care centres, prisons, hospital wards and kindergartens are not uncommon developed counties. In Australia it is neither given to contacts of index cases, nor to households in heavily infected large communities, as the prescribing information does not support ivermectin administration to contacts of index cases [31].

Permethrin resistance is widespread in other ectoparasites [32]. Evidence of increasing acaricide resistance leading to treatment failures has been reported (Table 1) [33–37]. *In vitro* sensitivity data of scabies mites from the last 10 years (Australian data) indicate that median survival times to leading acaricides (ivermectin and permethrin) have increased 2–3 fold [33, 34]. Treatment failure of permethrin as a scabicide in Indigenous communities in Australia (following MDA) and elsewhere has been documented and it is the slowest-acting acaricide (*in vitro*) in the Northern Territory, Australia [33, 38]. Permethrin resistance to scabies mites has been confirmed in an animal model and its likely resistance mechanism has also been documented [37, 39]. Since the first documented case in Australia in 1994 [40], there have been reports of resistance of *Sarcoptes scabiei* to ivermectin *in vitro* and *in vivo,* including treatment failure in clinical trials [30, 34, 41–43]. MDAs programs that encounter poor compliance increase the risk of developing resistance and targeted treatment of index cases and contacts may be a better approach [44].

## Conclusions

- Availability of a ‘fool-proof’ diagnostic tool will enable the selective treatment of affected individuals, decrease the potential for escalating mite resistance, and reduce the need for mass treatment and the associated costs.
- Long-term adherence difficulties, safety and efficacy uncertainties in the young and elderly, and growing concerns over the development of resistance to classical scabicides, all signal the need to identify new treatment options for scabies (with greater levels of treatment compliance in MDA programs) to reduce the burden of infection in endemic settings and the morbidity and mortality associated with it.

## Abbreviations

MDA: Mass Drug Administration Programs
WHO: World Health Organisation
